# Improving the Aroma of Millets by Targeting the *Betaine Aldehyde Dehydrogenase 2* Gene: A Promising Approach for Popularising Millet Foods Worldwide

**DOI:** 10.2174/0113892029353221251120094444

**Published:** 2026-01-15

**Authors:** T.P. Ajeesh Krishna, Mathew Veena, Theivanayagam Maharajan, Jos T. Puthur, Stanislaus Antony Ceasar

**Affiliations:** 1 Division of Plant Molecular Biology and Biotechnology, Department of Biosciences, Rajagiri College of Social Sciences (Autonomous), Kochi, Kerala, 683 104, India;; 2 Plant Physiology and Biochemistry Division, Department of Botany, University of Calicut, Calicut University Campus, Malappuram, Kerala, 673 635, India

**Keywords:** 2-acetyl-1-pyrroline, *BADH2* gene, food and nutritional security, genome-editing, human health, millets

## Abstract

Millets are highly nutritious and traditional staple foods consumed by millions worldwide. However, their low palatability and limited aroma have restricted their broader acceptance in human diets. Aroma is a key trait influencing consumer preference, and in many crops, 2-acetyl-1-pyrroline (2AP) contributes significantly to fragrance and palatability. The *betaine aldehyde dehydrogenase 2* (*BADH2*) gene is a critical regulator of 2AP biosynthesis in crops. Targeted manipulation of *BADH2* in the metabolic pathway offers a promising strategy to enhance 2AP production in millets. Understanding the structure and function of BADH2 is essential for improving aroma traits, and genome editing (GE) approaches present a viable avenue for functional enhancement. In this review, we highlight the significance of 2AP and its metabolic pathways in crops and provide insights into the structural and functional features of BADH2. We mined putative BADH proteins from foxtail millet, finger millet, and sorghum using the rice OsBADH2 sequence as a reference, and analyzed their physicochemical and protein characteristics *via in silico* approaches. Furthermore, we discuss potential functional motif modifications of BADH2 in millets to enhance 2AP production through GE strategies. This review offers a comprehensive perspective on engineering BADH2 functional motifs to develop fragrant millet varieties. These insights could accelerate millet improvement and support the global promotion and adoption of millet-based foods.

## 1 INTRODUCTION

Food scarcity and malnutrition remain major challenges in developing countries due to the rapidly growing global population. The world population is projected to reach 10 billion by 2050 [1], necessitating a 60–70% increase in food production to meet global demand [2]. Importantly, this increased production must ensure high-quality crops to prevent widespread malnutrition. Expanding arable land is increasingly difficult, and unpredictable climate change poses additional threats to sustainable crop production. Cereals are the primary staple for people worldwide, providing more than 50% of daily caloric intake [3], but they are inherently low in bioavailable micronutrients [4]. Beyond major cereals, there is an urgent need to promote lesser-known and underutilized crops to address future food and nutrition challenges.

Millets are small-seeded, nutritious coarse cereals that serve as staple foods for millions in Asia and Africa. They are highly climate-resilient and can be cultivated in arid and semi-arid regions with limited water availability [5-7]. Millets include sorghum (*Sorghum bicolor* L.), pearl millet (*Cenchrus americanus* (L.) Morrone), finger millet (*Eleusine coracana* (L.) Gaertn), foxtail millet (*Setaria italica* (L.) Beauv), little millet (*Panicum sumatrense* Roth. ex Roem. and Schult), proso millet (*Panicum miliaceum* L.), kodo millet (*Paspalum scrobiculatum* L.), barnyard millet (*Echinochloa esculenta* (A. Braun) H. Scholz), teff (*Eragrostis tef* (Zucc.) Trotter), fonio (*Digitaria exilis* Stapf and *D. iburua* Stapf), job’s tears (*Coix lacryma-jobi* L.), guinea millet (*Brachiaria deflexa* (Schumach.) C.E.Hubb. ex Robyns), and browntop millet (*Brachiaria ramosa* (L.) Stapf) [2, 8]. Among these, sorghum and pearl millet are categorized as major millets, while the others are considered minor millets.

Despite their nutritional superiority over major cereals like rice, wheat, and maize [9-16], millet consumption remains low, with only an estimated 1.2 billion people including it in their diet [17]. A major factor limiting millet consumption is low palatability and the absence of an appealing aroma. Enhancing millet aroma and grain quality can significantly boost their global popularity, encourage regular dietary consumption, and improve human health outcomes.

In recent years, considerable attention has been given to improving grain quality traits using genome-based approaches. The success of aromatic rice varieties such as Basmati and Jasmine demonstrates the market potential of fragrant grains [18]. The key molecule responsible for rice aroma, 2-acetyl-1-pyrroline (2AP), was identified by Buttery *et al.* in 1982 [19]. In rice, 2AP accumulation is primarily regulated by the betaine aldehyde dehydrogenase 2 (BADH2) gene, with loss-of-function mutations or reduced expression leading to fragrance [20-23]. For instance, Hui *et al.* [24] reported that *BADH2* gene mutations enhance rice grain aroma without compromising other quality traits. Similarly, an amino acid substitution (Gly364Asp) in GmBADH2 confers fragrance in soybeans [25]. Manipulating key functional residues in BADH2 may therefore reduce enzyme activity and increase 2AP production. Understanding these functional residues in millet BADH2 proteins is critical for aroma improvement, necessitating high-resolution structural insights.

Whole-genome sequencing (WGS) is indispensable for understanding gene organization, mining proteomic resources, and performing comparative studies of BADH2 in millets [26]. Currently, the genomic sequences of six millets, sorghum, pearl millet, foxtail millet, finger millet, proso millet, and barnyard millet, are available (Table **1**). However, only three annotated genomes (sorghum, foxtail millet, and finger millet) are present in the Phytozome database, limiting opportunities for BADH2 mining and functional validation in other millets. Structural and functional characterization of BADH2 in millets remains largely unexplored. Previously, Baicharoen *et al.* [27] modeled the 3D dimeric structure of OsBADH2 (UniProtKB: O24174) using PsAMADH2 (PDB: 3IWJ) as a template. Comparative analysis with millet BADH2 proteins could provide valuable insights for genome-editing (GE)-based functional modifications aimed at enhancing 2AP content.

In this review, we systematically searched PubMed Central, ScienceDirect, Google Scholar, Scopus, and Web of Science for literature on BADH2 and 2AP biosynthesis in millets, with no time restriction. We mined BADH gene and protein sequences from sorghum, foxtail millet, and finger millet using rice OsBADH1 (UniProt: O24174) and OsBADH2 (UniProt: Q84LK3) as references. Structural and functional analyses of millet BADH2 proteins were conducted *in silico* using Clustal Omega (https://www.ebi.ac.uk/jdispatcher/msa/clustalo?stype=protein), and physicochemical parameters were evaluated *via* the ProtParam tool (https://web.expasy.org/protparam/). We also review the role of BADH2 in the 2AP biosynthesis pathway and discuss genome-editing strategies for enhancing aroma in millets.

Overall, this article provides insights into improving 2AP content in millets to enhance palatability, increase global consumption, and position millets as a key crop for future food and nutritional security worldwide.

## 2 BIOSYNTHETIC PATHWAY OF 2AP IN CROP PLANTS

The regulation of 2-acetyl-1-pyrroline (2AP) biosynthesis occurs primarily at the genetic level. Identification and manipulation of key genes controlling this metabolic pathway can enhance the production of desirable aroma compounds [28]. 2AP is a ketone responsible for the distinct fragrance of aromatic rice varieties such as Basmati and Jasmine, often described as popcorn-like. Understanding the genes involved in 2AP biosynthesis is crucial for increasing its content in millets through genome-based approaches.

**Fig. (1):**
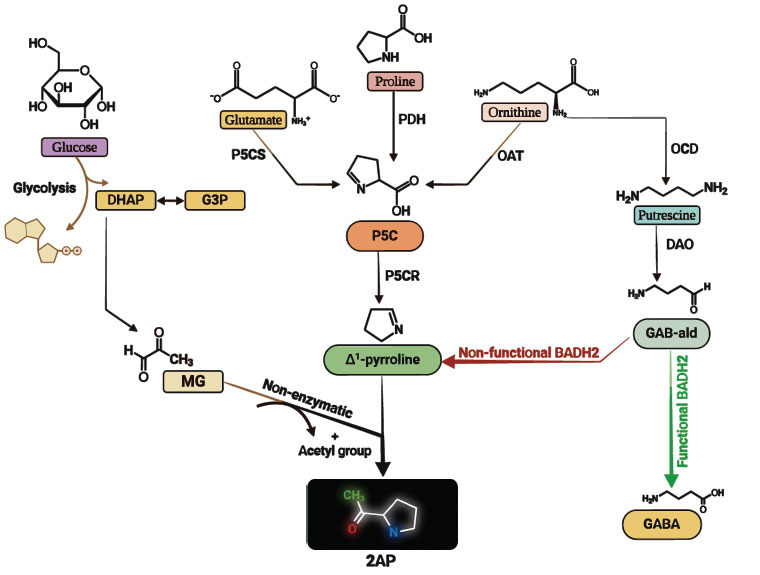
**A schematic illustration of biosynthesis pathway of 2AP.** Two different pathways can produce 2AP. Route 1: A pathway independent of BADH2. Proline, glutamate, and ornithine are transformed into Δ^1^‐pyrolline‐5‐carboxylic acid (P5C) by the enzymes proline dehydrogenase (PDH), Δ^1^‐pyrolline‐5‐carboxylic acid synthetase (P5CS), and ornithine aminotransferase (OAT), respectively. The P5C is transformed into Δ^1^-pyrroline by the enzyme pyrroline-5-carboxylate reductase (P5CR). The Δ^1^-pyrroline is an immediate precursor of 2AP. Route 2: Pathway dependent on BADH2. Ornithine is transformed into putrescine by ODC and then into gamma-aminobutyric acid (GAB-ald) by diamine oxidase (DAO). This GAB-ald has two possible outcomes. Functional betaine aldehyde dehydrogenase 2 (BADH2) converts it into gamma-aminobutyric acid (GABA), while non-functional BADH2 causes it to cyclize to generate Δ^1^-pyrroline. Δ^1^-pyrroline, produced *via* BADH2-dependent and independent pathways, is transformed into 2AP spontaneously when an acetyl group is added to it from methylglyoxal (MG). The image was created with www.BioRender.com.

The 2AP biosynthetic pathway has been extensively studied (Fig. **1**). 2AP is derived from the heterocyclic molecule Δ^1^-pyrroline, which originates from amino acids such as proline, glutamic acid, and ornithine [29, 30]. These precursors are converted into Δ^1^-pyrroline-5-carboxylic acid (P5C) through the enzymatic activity of proline dehydrogenase (PDH), Δ^1^-pyrroline-5-carboxylate synthetase (P5CS), and ornithine aminotransferase (OAT), using proline, glutamate, and ornithine as substrates, respectively [20, 31, 32].

Glutamate is first converted to P5C *via* an intermediate compound, glutamate semialdehyde (GSA). P5C subsequently cyclizes to form Δ^1^-pyrroline [33, 34]. The addition of an acetyl group from methylglyoxal (MG) to Δ^1^-pyrroline leads to 2AP formation. MG can also directly convert P5C into 2AP through a non-enzymatic route [35-38].

Modulating the levels of these intermediates can influence 2AP production and thus fragrance. MG is produced from triose phosphates generated in glycolysis, the Calvin cycle, protein catabolism, and fatty acid metabolism [39, 40]. Ornithine can also be synthesized from proline and arginine. Putrescine is formed *via* the enzymatic activity of ornithine decarboxylase (ODC), and ornithine diamine oxidase (DOA) catalyzes the conversion of putrescine to gamma-aminobutyraldehyde (GAB-ald).

The BADH2 enzyme plays a critical role in this pathway: when functional, BADH2 converts GAB-ald to gamma-aminobutyric acid (GABA), thereby inhibiting 2AP biosynthesis. In contrast, in the absence or loss-of-function state of BADH2, GAB-ald spontaneously cyclizes to form Δ^1^-pyrroline, which is then converted to 2AP *via* acetylation by MG (Fig. **1**) [31]. BADH2 is a recessive gene that suppresses 2AP production. Mutations or down-regulation of BADH2 have been shown to enhance 2AP accumulation in rice [22, 41].

Targeting the BADH2 gene in millets, through mutation, silencing, or genome-editing approaches, represents a promising strategy to increase 2AP levels and improve the aroma and palatability of millet grains.

## 3 IMPORTANCE OF ENHANCING THE 2AP IN MILLETS

Sensory qualities, including taste, flavor, and aroma, are key determinants of grain quality and consumer preference [42, 43]. Enhancing these attributes in millets can increase their global appeal and demand. Premium grains such as Basmati rice from India and Jasmine rice from Thailand exemplify how superior sensory qualities contribute to worldwide popularity [44]. In contrast, the relatively low palatability and aroma of millets have limited their consumption globally.

Beyond sensory appeal, millets are versatile crops with the potential to address future global food, nutritional, health, and economic challenges. Their cultivation aligns with the United Nations Sustainable Development Goals (SDGs), particularly those related to food and nutritional security [26]. Millets are nutritionally rich, providing carbohydrates, dietary fiber, energy, essential fatty acids, proteins, B vitamins, minerals, and phytochemicals. They are especially abundant in mineral nutrients such as calcium, iron, magnesium, potassium, and zinc, which help prevent malnutrition and non-communicable diseases [45-47].

Finger millet, for instance, is a notable source of calcium [48], which supports fetal skeletal growth, increases birth weight, and helps prevent preeclampsia in pregnant women [49, 50]. Magnesium, also present in significant amounts [51], contributes to reducing hyperlipidemia and lowering the risk of heart disease [52, 53]. Millets are naturally gluten-free, making them suitable for individuals with celiac disease, an autoimmune disorder characterized by small intestinal mucosal injury caused by gluten [54]. Consequently, millets offer promising health benefits for people with celiac disease, ischemic heart disease, obesity, dyslipidemia, type 2 diabetes mellitus, and other metabolic disorders [55-58].

In addition to their nutritional value, millets are highly climate-resilient, ensuring sustainable food security. They can thrive in diverse environmental conditions, particularly in semi-arid regions, and can achieve high yields within short growing seasons [59-61]. Certain millets also exhibit excellent storability, remaining pest-free for several years [62].

Promoting millet cultivation and consumption can contribute to a sustainable and healthy food system. Enhancing the aromatic and sensory qualities of millets through metabolic engineering or genome-based approaches can further increase their acceptance worldwide. This improvement has the potential not only to boost daily consumption but also to elevate their economic value, incentivizing production and supporting global nutritional security.

## 4 STRUCTURAL AND FUNCTIONAL INSIGHTS OF BADH2 IN CROP PLANTS

Betaine aldehyde dehydrogenase (BADH) belongs to the aldehyde dehydrogenase (ALDH) family, which includes NAD(P)+-dependent enzymes that catalyze the conversion of various intermediate aldehydes into their corresponding carboxylic acids [63]. This family is highly conserved across a wide range of organisms, including plants. BADH genes serve multifunctional roles in crop plants. For example, the rice genome encodes two homologs of the BADH protein: OsBADH1 (LOC_Os04g39020) and OsBADH2 (LOC_Os08g32870), sharing 75.94% sequence similarity [27]. OsBADH1 is primarily involved in modulating abiotic stress responses [64, 65], whereas OsBADH2 plays a key role in the biosynthesis of 2-acetyl-1-pyrroline (2AP), the major aromatic compound in rice [27, 66].

Understanding the structural and functional features of BADH2 is crucial for regulating its catalytic activity, which could directly influence 2AP synthesis in crop plants. However, the crystal structure of BADH2 in crops is currently unavailable. BADH proteins are closely related to aminoaldehyde dehydrogenase (AMADH) in plants, sharing ~76% sequence similarity [27]. Crystal structures of pea AMADH proteins, PsAMADH1 (PDB: 3IWK) and PsAMADH2 (PDB: 3IWJ), are known and exist as dimers [67]. Each subunit contains three primary domains: a NAD+-binding domain, an oligomerization domain, and a substrate-binding domain. Their enzymatic mechanisms have been predicted based on these structures [67].

In rice, OsBADH2 also forms a dimer, with each subunit comprising NAD+-binding, oligomerization, and substrate-binding domains. Previous simulation and mutagenesis studies have identified several residues critical for substrate recognition and binding, including Asn162, Tyr163, Met167, Trp170, Glu260, Trp288, Ser295, Trp420, Cys453, and Trp459 [68-70]. Among these, Asn162, Glu260, and Cys294 are particularly important: Glu260 and Cys294 are involved in hemithioacetal formation, while Asn162 stabilizes reaction intermediates. These residues are highly conserved across BADH2 proteins in various crops [27].

Baicharoen *et al.* [27] recently performed homology modeling of OsBADH2 using PsAMADH2 as a template (PDB: 3IWJ), providing structural and dynamic insights that are valuable for enhancing rice fragrance. Protein sequence variations between OsBADH2 and PsAMADH2 can affect conserved functional residues, as observed in other protein families such as ZIP metal transporters, where sequence variation led to differences in functional residues involved in metal binding and transport [71].

Given the central role of BADH2 in 2AP biosynthesis, detailed structural and functional characterization in millets is essential. Such insights would allow targeted engineering of key residues, enabling genome-editing approaches to enhance 2AP production and improve the aromatic quality of millet grains.

## 5 PROTEIN FEATURES OF BADH IN MILLETS

Using the OsBADH1 (UniProt ID: O24174) and OsBADH2 (UniProt ID: Q84LK3) protein sequences as references, we mined putative BADH proteins from foxtail millet (Seita.6G151100 and Seita.7G127300), finger millet (ELECO.r07.4AG0314620, ELECO.r07.4BG0345670, ELECO.r07.8AG0631100, and ELECO.r07.8BG0660040), and sorghum (Sobic.006G109500 and Sobic.007G130800) from the Phytozome database (https://phytozome-next.jgi.doe.gov/). Probable gene names for the mined millet BADH proteins were assigned based on their similarity to the rice OsBADH proteins (Table **2**). The complete millet BADH protein sequences are provided in Supplementary File **1**.

The analyzed millet BADH proteins are composed of 506–507 amino acids, with molecular weights ranging from 54.73 to 55.06 kDa. The overall protein features of millet BADHs are summarized in Table **2**. While BADH genes have previously been reported in foxtail millet and sorghum [72, 73], this is the first report identifying BADH genes from the finger millet genome. Importantly, the protein features of millet BADH1 and BADH2 closely resemble those of rice OsBADH1 and OsBADH2 (Table **2**).

Understanding the functional motif sequences of millet BADHs is crucial for potential transcriptional regulation or functional mutation of these genes. Bradbury *et al.* [74] reported that the functional OsBADH2 protein contains two conserved motifs (VSLELGGKSP and EGCRLGSVVS) and a critical cysteine (Cys) residue, located 28 amino acids downstream of the VSLELGGKSP motif. These motifs and the Cys residue are highly conserved across the ALDH family and are essential for the functional activity of OsBADH2 [74].

**Fig. (2):**
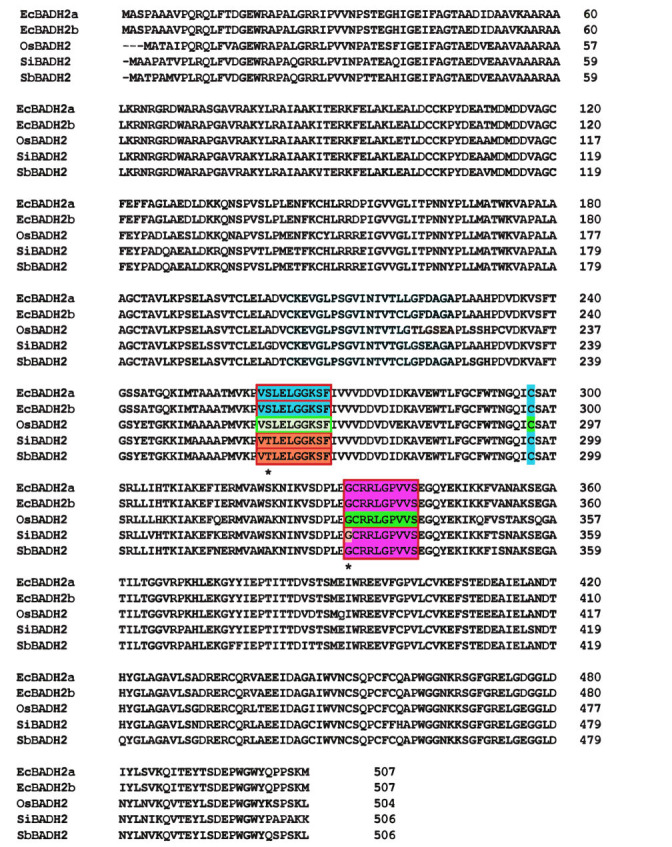
**Clustal alignment of the amino acid sequences of the BADH2 protein from millets and rice.** The OsBADH2 sequence was used as a template. The highlighted (green) sequences, including the VSLELGGKSP and EGCRLGSVVS motifs, as well as a Cys amino acid residue, are considered essential for the functional activity of OsBADH2 in rice [74]. In millets, the homologous residues corresponding to the functional motifs and the Cys residue are highlighted in red, turquoise, and pink, respectively. The analysis was done with Clustal Omega Multiple Sequence Alignment online tool and edited with power point.

Clustal Omega analysis revealed that these motifs and the Cys residue are highly conserved in finger millet, foxtail millet, and sorghum BADH2 sequences (Fig. **2**). In particular, the BADH2 sequences of finger millet and sorghum are fully conserved with the OsBADH2 motifs and Cys residue. Interestingly, SiBADH2 (foxtail millet) shows single amino acid variations: VSLELGGKSP → VTLELGGKSP (Ser → Thr) and EGCRLGSVVS → DGCRLGPVVS (Glu → Asp), which may alter its functional activity. Similar single amino acid variations were also observed in SbBADH2 (sorghum) (Fig. **2**). The functinal motif sequences of all millet BADH2 genes are provided in Supplementary File **2**.

A precedent for functional impact of motif variations exists in soybean: comparison of GmBADH2 between fragrant (Kaori) and non-fragrant (CM60) varieties revealed a non-synonymous SNP (Gly → Asp, GGC → GAC) in the EGCRLGPIVS motif, which results in loss of functional activity in Kaori and production of aroma [25]. By analogy, the amino acid variations observed in millet BADH2 motifs may similarly influence 2AP biosynthesis and fragrance.

These findings highlight the need for in-depth molecular studies to characterize the functional roles of these amino acid residues in millet BADH2 proteins. Such studies will provide crucial insights for targeted gene manipulation or genome-editing approaches aimed at enhancing 2AP content and improving the aromatic quality of millet grains.

## 6 OPPORTUNITY FOR GENOME EDITING WITH CRISPR-CAS TOOLS FOR ENHANCING THE 2AP CONTENT IN MILLETS

Recent advancements in GE technology have made it possible for scientists to more accurately and effectively alter the genes of a wide range of organisms, including plants [75, 76]. GE tools help to remove, insert, or replace desirable nucleotide bases in the targeted genome [77, 78]. This allows the creation of functional or non-functioning states of the targeted genes, which can lead to improvements in plant traits. Meganucleases (MgNs), zinc finger nucleases (ZFNs), and transcription activator-like effector nucleases (TALENs) have been widely used for GE over the past several years [79-81]. These tools induce DNA double-strand breaks (DSBs), which stimulate error-prone non-homologous end joining (NHEJ) or homology-directed repair (HDR) at specific genomic regions. However, MgNs, ZFNs, and TALENs have some limitations and require high-cost, labor-intensive procedures [82].

By contrast, the clustered regularly interspaced short palindromic repeat (CRISPR)/CRISPR-associated protein 9 (Cas9) system is a simple, site-directed mutagenesis strategy based on guide RNA (gRNA) [83]. It has emerged as an efficient, robust, and user-friendly tool [84]. Further progress in the CRISPR/Cas9 system, such as nuclease-dead mutants of Cas9 (dCas9), which lose endonuclease activity but retain DNA recognition ability, offers gene interference (CRISPRi) and activation (CRISPRa) [85]. Base editing (BE) and prime editing (PE) are further expanded CRISPR/Cas systems that can create transition and transversion mutations, as well as small insertions or deletions [86, 87]. Today, the CRISPR/Cas system is widely used as a cutting-edge biotechnological tool for crop improvement [88, 89]. In addition to crop improvement, the CRISPR/Cas9 system is a promising approach for metabolic engineering [90]. It can help with transcriptional regulation or functional mutation of genes in metabolic pathways, ultimately contributing to the synthesis of desirable metabolites in crop plants.

Using the CRISPR/Cas system to manipulate the BADH2 gene will help improve the synthesis of 2AP in millet seeds. So far, very little effort has been made to enhance 2AP content in millet seeds using CRISPR/Cas9. Recently, Zhang and co-workers [72] reported that knockout of the SiBADH2 gene using CRISPR/Cas9 enhanced 2AP in mature foxtail millet seeds and leaves. This study revealed that functional loss of SiBADH2 leads to a popcorn-like fragrance in previously non-aromatic foxtail millet [72]. Furthermore, CRISPR/Cas9-targeted knockout of SbBADH2 enhanced 2AP accumulation in sorghum seeds and leaves [73]. The CRISPR/Cas9 system has also been successfully used to enhance 2AP in other crops (Table **3**). For example, knockout of OsBADH2 increased 2AP content in mutated non-aromatic rice lines (*Osbadh2*) [91]. Chen and co-workers [92] developed single (*Ntbadh1a, Ntbadh1b, Ntbadh2a*, or *Ntbadh2b*) and double (*Ntbadh1a-Ntbadh1b* and *Ntbadh2a-Ntbadh2b*) mutant tobacco lines using CRISPR/Cas9. The popcorn-like fragrance was noticeable in leaves of the double mutant (*Ntbadh2a-Ntbadh2b*) lines, whereas single mutants or double mutants of Ntbadh1a–Ntbadh1b did not produce fragrance [92]. These studies indicate that functional loss of BADH2 homologs is essential for creating the popcorn-like aroma.

**Fig. (3):**
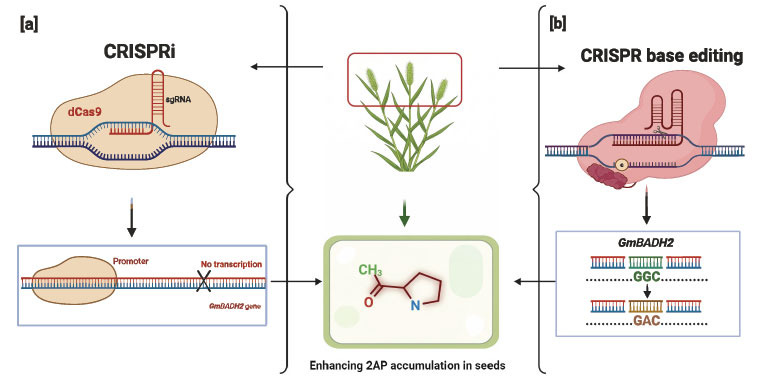
**Application of CRISPR/Cas variants for improving 2AP synthesis in crops.** (**a**) Suppression of GmBADH2 transcription *via* CRISPRi may enhance 2AP accumulation in millet seeds. (**b**) CRISPR/Cas base editor for editing functional residues of *GmBADH2*. The coding sequence required for the *GmBADH2* gene’s mutagenesis of specific amino acids (GGC to GAC) is projected based on the original gene sequence for the *GmBADH2* gene. The targeted base changeto A) results in a fuaional mutation in the Gmthe BADH2 gene, imimpairing itsenhancing is oenhancing. The image was created with www.BioRender.com.

Advances in CRISPR/Cas base editing (BE) allow precise modification of single-nucleotide variants at specific loci in target DNA [93]. Mutation, substitution, or deletion of functional residues in the motif may alter gene functions. For example, the single amino acid variation in the functional motif (EGCRLGPIVS to EGCRLDPIVS) of GmBADH2 is responsible for fragrance in soybeans [25]. Manipulation of BADH2 functional motifs can lead to loss of function, enhancing 2AP synthesis (Fig. **3**). Lilay and co-workers [94] demonstrated that substitution of critical cysteine and histidine residues with alanine in the zinc-sensor motif of transcription factor bZIP19 abolished zinc-binding activity, showing the importance of motif residues in functional activity. We anticipate that similar CRISPR/Cas-mediated alterations in BADH2 functional motifs can enhance 2AP synthesis in millets. We predicted the functional motifs of millet BADH2 genes using OsBADH2 as a template (Table **2**, Fig. **2**), and the sequences are provided in Supplementary File **2**.

Beyond functional motifs, BADH2 has other residues involved in NAD+-binding, oligomerization, and substrate-binding. Alteration of these residues may reduce enzyme activity and contribute to higher 2AP synthesis, creating aroma in millets. Ashokkumar and co-workers [91] generated new allelic variations of OsBADH2 *via* CRISPR/Cas9, showing that amino acid changes can enhance aroma in non-aromatic rice varieties.

Future research should focus on organ-specific (seed) knockout of *BADH2* genes in millets. This approach could increase 2AP content specifically in seeds while preserving GABA levels in other tissues, which are important for stress tolerance. Identification and validation of seed-specific promoters are critical for this purpose. For instance, pF128 is a seed-specific promoter in foxtail millet, with mRNA expressed only in immature and mature seeds [95]. Seed-specific promoters should be utilized to knock out BADH2 in millets using CRISPR/Cas, maximizing 2AP accumulation in seeds.

Although CRISPR/Cas has been widely applied in rice, its application in millets is still limited due to challenges such as inefficient transformation and regeneration protocols and the limited availability of well-annotated millet genomes [96]. Nonetheless, CRISPR/Cas offers a promising approach to popularize millet consumption by enhancing 2AP content, and researchers should focus on further developing this strategy for millet improvement (Fig. **3**).

## 7 FUTURE PERSPECTIVES FOR POPULARISING MILLET FOODS WORLDWIDE

Climate change and overpopulation threaten to reverse the progress achieved so far in the fight against food and nutrition security. In this context, promoting suitable foods and nutritionally rich crops is critical worldwide. Consequently, ensuring future food and nutritional security has become a major research priority. Millets are increasingly recognized as a crop of the future for achieving food and nutritional security. They are resilient, able to thrive under diverse climates, including areas with limited rainfall and unfavorable soils. Their adaptability makes them a durable and environmentally sustainable choice for cultivation across various regions. In addition, millets are nutritionally superior to major cereal crops. Beyond traditional uses, millets can be processed into a variety of food products, such as millet cookies, noodles, and wine. Improving millet grain quality, therefore, has the potential to significantly contribute to future food and nutritional security. Sensory traits such as smell, taste, and texture primarily attract consumers and strongly influence their willingness to include millets in their daily diet.

Scientists need to focus on improving these sensory traits in millets. 2-acetyl-1-pyrroline (2AP) is a chemical compound responsible for the distinctive aroma of certain foods. The *BADH2* gene plays a key role in 2AP biosynthesis in rice, and its non-functional state is responsible for fragrance. The function of *BADH2* has been validated through CRISPR/Cas9-mediated knockout studies. However, beyond aroma, other sensory-related traits also need attention in millets. For instance, amylose content is a key determinant of rice palatability. Rice with low amylose content (<12%) exhibits a soft and glutinous texture with excellent eating quality [97]. Similarly, amylopectin content significantly affects texture and stickiness; higher amylopectin produces softer, stickier grains, whereas lower amylopectin yields firmer, less sticky rice. In millets, amylose and amylopectin typically constitute 20–30% and 70–80% of total starch content, respectively [98].

The functional activity of waxy (Wx) genes is crucial for amylose synthesis in rice grains [99]. The Wx gene encodes granule-bound starch synthase (GBSS), which synthesizes amylose [100, 101], and functional mutations in Wx can reduce amylose content in rice [102]. Amylopectin biosynthesis is primarily regulated by genes encoding starch synthases (SS), starch branching enzymes (SBE), and debranching enzymes (DBE) [103]. Therefore, identification and characterization of key genes controlling amylose and amylopectin biosynthesis are essential for modifying starch composition in millet grains.

Additionally, various phytochemical compounds naturally present in millet influence taste, aroma, and texture. While these compounds provide nutritional and physiological benefits, some may impart undesirable flavors or mouthfeel, limiting the appeal of millet-based foods. Consequently, manipulation of genes responsible for improving smell, taste, and texture in millets is a high-priority research area.

In the coming years, significant progress is expected in using CRISPR-based strategies to fine-tune these sensory traits in millets. This may involve knockout or base editing of negative regulators or activation of beneficial alleles that govern aroma, taste, and grain softness. By integrating high-throughput phenotyping with advanced genomic and metabolomic tools, breeders and molecular biologists will be better positioned to develop millet varieties that align with consumer preferences. Ultimately, such innovations will enhance the palatability of millet while also boosting its market potential as a climate-resilient and nutritious staple in both traditional and modern food systems.

## CONCLUSION

Millet consumption is markedly lower than that of major cereal crops such as rice, wheat, and maize. Although millets serve as an essential staple in arid and semi-arid regions, they account for less than 2% of global cereal utilization. One of the main challenges is their limited palatability, which reduces their appeal. Improving the taste of millets could enhance their utilization in the food industry and increase the consumption of millet-based products worldwide. To date, no naturally fragrant millet varieties have been reported, except for sorghum. Enhancing 2-acetyl-1-pyrroline (2AP) accumulation in millet grains could significantly improve their palatability.

Tissue-specific promoters are active only in particular cell types, making the selection of a seed-specific promoter a critical step in regulating *BADH2* genes in millet seeds. Using seed-specific promoters in genome-editing (GE) approaches can help enhance 2AP specifically in seeds, avoiding potential effects in other tissues. Currently, there is limited information available on the *BADH2* gene in millets, highlighting the need for more research focused on seed-specific knockout of BADH2.

In this review, we highlight the structural and functional features of BADH2 in millets. We mined the EcBADH proteins (EcBADH1a, EcBADH1b, EcBADH2a, and EcBADH2b) from the finger millet genome sequence and analyzed their protein features. This represents the first report on the *in silico* analysis of BADH genes in finger millet. Furthermore, this is the first comprehensive review providing insight into the functional motifs of BADH2 in millets. This information offers a foundation for developing new fragrant millet varieties *via* genome-editing approaches. Manipulating the functional state of BADH2 in millet could ultimately help popularize millet foods worldwide, with fragrance contributing to future food and nutritional security.

Beyond fragrance, researchers should also focus on improving other sensory traits in millets, including taste and texture, to increase their appeal and promote their incorporation into daily diets.

## Figures and Tables

**Table 1:** Current ststus of genome sequence available for millets.

**Millet Category**	**Name of Millet**	**Genotype**	**% of Genome Coverage**	**Genome Size (Mb/Gb)**	**Total Number of Genes**	**References**	**Annotated Genome**
Major millets	Sorghum	BTx623	⁓98%	~730 Mb	28375	[104]	Avilable
	Pearl millet	Tift 23D2B1-P1-P5	⁓90%	~1.79 Gb	69398	[105]	Nil
Minor millets	Foxtail millet	cv. Zhang gu × A10	⁓80%	⁓400 Mb	24000 - 29000	[106]	Avilable
		cv. Zhang gu × A2	⁓86%	⁓423 Mb	38801	[107]	
	Finger millet	ML-365	⁓82%	1196 Mb	85243	[108]	Avilable
		PR 202	78.2%	1189 Mb	62348	[109]	
	Proso millet	Landrace (Ac. No: 00000390)	91.9%	~923 Mb	55930	[110]	Nil
	Barnyard millet	STB08	90.7%	1.27 Gb	108771	[111]	Nil

**Note:** The millet category, millet name, genotype used for WGS, % of genome coverage, genome size, and the total number of genes are provided with reference. The status of annotated millet genome also mentioned.

**Table 2:** Details of physicochemical and protein features of putative BADH proteins of foxtail millet, finger millet, sorghum, and rice.

**Millet Name**	**Gene ID**	**Predicted Gene Name**	**Protein Length**	**Blast E-Value**	**MW (kDa)**	**p*I* value**	**Probable Functional Motif**
Foxtail millet	Seita.7G127300	*SiBADH1*	506	0	55.03	5.21	VTLELGGKSP and DGCRLGPVVS
	Seita.6G151100	*SiBADH2*	506	0	55.01	5.21	VTLELGGKSP and DGCRLGPVVS
Finger millet	ELECO.r07.4AG0314620	*EcBADH1a*	506	0	54.77	5.63	VSLELGGKSP and EGCRLGSVVS
	ELECO.r07.4BG0345670	*EcBADH1b*	506	0	54.73	5.44	VSLELGGKSP and EGCRLGSVVS
	ELECO.r07.8AG0631100	*EcBADH2a*	507	0	54.76	5.31	VSLELGGKSP and EGCRLGPVVS
	ELECO.r07.8BG0660040	*EcBADH2b*	507	0	54.79	5.31	VSLELGGKSP and EGCRLGPVVS
Sorghum	Sobic.006G109500	*SbBADH1*	507	0	55.06	5.94	VSLELGGKSP and EGCRLGSVVS
	Sobic.007G130800	*SbBADH2*	506	0	54.91	5.16	VTLELGGKSP and EGCRLGPVVS
Rice	LOC_Os04g39020	*OsBADH1*	506	0	54.64	6.63	VSLELGGKSP and EGCRLGSVVS
	LOC_Os08g32870	*OsBADH2*	504	0	54.68	5.36	VSLELGGKSP and EGCRLGPVVS

**Note:** The protein sequences were mined from the Phytozome (https://phytozome-next.jgi.doe.gov/) database. The name of the plant, gene ID, predicted gene name, protein length, E-value, MW (kDa), p*I* value, and probable functional motif are provided. The protein sequences are provided in Supplementary File **1**.

**Table 3:** List of mutation studies of *BADH2* genes *via* CRISPR/Cas system for enhancing 2AP in various plants. Details on crop plants, target gene, CRISPR editing type, Cas9 and SgRNA promoters, method of construct delivery, plasmid, gene function, and results were included, with references.

**Name of the Crop**	**Targeted Gene Name**	**Editing Type**	**Cas9 Promoter**	**SgRNA Promoter**	**Method of Delivery**	**Plasmid Used**	**Function of a Gene**	**Observation**	**References**
Rice	*OsBADH2*	CRISPR/Cas9- mediated mutagenesis	CaMV 35S	*OsU3*	*Agrobacterium*-mediated transformation	pRGEB31	Responsible for 2AP synthesis	Edited non-aromatic mutant (*Osbadh2*) rice plants showed a strong aroma	[91]
	*OsBADH2*	CRISPR/Cas9- mediated mutagenesis	CaMV 35S	*OsU3*	*Agrobacterium*-mediated transformation	pCXUN	Responsible for 2AP synthesis	Exhibited a strong aroma in the mutant rice plants	[112]
	*OsBADH2*	CRISPR/Cas9- mediated mutagenesis	CaMV 35S	*OsU6*	*Agrobacterium*-mediated transformation	VK005-1	Responsible for 2AP synthesis	Exhibited a strong aroma in the mutant (*Osbadh2*) rice plants	[24]
Maize	*ZmBADH2a* and *ZmBADH2b*	CRISPR/Cas9- mediated mutagenesis	Ubiquitin	*ZmU6*	*Agrobacterium*-mediated transformation	p0195	Responsible for 2AP synthesis	Double-mutant (*Zmbadh2a/Zmbadh2b*) maize plants have a strong aroma	[113]
	*ZmBADH2-1* and *ZmBADH2-2*	CRISPR/Cas9- mediated mutagenesis	Ubiquitin	*ZmU6*	*Agrobacterium*-mediated transformation	pTiBo542	Responsible for 2AP synthesis	Edited plants (*Zmbadh2-1/Zmbadh2-2*) showed a strong aromatic flavor	[114]
Tobacco	*NtBADH2a* and *NtBADH2b*	CRISPR/Cas9- mediated mutagenesis	Ubiquitin	*OsU3*	*Agrobacterium*-mediated transformation	pOREU3TR	Responsible for 2AP synthesis	A popcorn-like scent is observed in the double-mutant (*Ntbadh2a-Ntbadh2b*) tobacco plants	[92]
Foxtail millet	*SiBADH2*	CRISPR/Cas9- mediated mutagenesis	Ubiquitin	*OsU6a*	*Agrobacterium*-mediated transformation	pBR322	Responsible for 2AP synthesis	A popcorn-like fragrance was observed in the non-aromatic (*Sibadh2*) mutant foxtail millet plants	[72]
Sorghum	*SbBADH2*	CRISPR/Cas9- mediated mutagenesis	Ubiquitin	*OsU6a*	*Agrobacterium*-mediated transformation	pBR322	Responsible for 2AP synthesis	Edited mutant plants showed a higher aromatic smell in seeds and leaves	[73]

## LIST OF ABBREVIATIONS

2AP: 2-acetyl-1-pyrroline
ALDH: Aldehyde Dehydrogenase
BADH2: Betaine Aldehyde Dehydrogenase 2
BE: Base-editing
CRISPR/Cas9: Clustered Regularly Interspaced Short Palindromic Repeat-associated Protein 9
CRISPRa: CRISPR Activation
CRISPRi: CRISPR Interference
DBE: Debranching Enzymes
DHAP: Dihydroxyacetone Phosphate
DOA: Ornithine Diamine Oxidase
DSBs: Double-strand Breaks
G3P: Glyceraldehyde3-phosphate
GAB-ald: Gamma-aminobutyaldehyde
GABA: Gamma-aminobutyric Acid
GBSS: Granule-bound Starch Synthase
GE: Genome-editing
gRNA: guided RNA
GSA: Glutamate Semi-aldehyde
HDR: Homology-directed Repair
MG: Methylglyoxal
MgNs: Meganucleases
NAD: β-nicotinamide Adenine Dinucleotide
NHEJ: Non-homologous End Joining
OAT: Ornithine Aminotransferase
ODC: Ornithine Decarboxylase
P5C: Δ^1^‐pyrolline‐5‐carboxylic Acid
P5CR: Pyrroline-5-carboxylate Reductase
P5CS: Δ^1^‐pyrolline‐5‐carboxylic Acid Synthetase
PDH: Proline Dehydrogenase
PE: Prime-editing
SBE: Starch Branching Enzymes
SDGs: Sustainable Development Goals
SS: Starch Synthases
TALENs: Transcription Activator-like Effector Nucleases
TF: Transcription Factor
ZFNs: Zinc Finger Nucleases
ZIP: Zinc-regulated, Iron-regulated Transporter-like Proteins
