# Supplementary material for: Improving the Aroma of Millets by Targeting the Betaine Aldehyde Dehydrogenase 2 Gene: A Promising Approach for Popularising Millet Foods Worldwide
Source: Curr Genomics. 2026 Jan 15;26(6):530–43. doi: 10.2174/0113892029353221251120094444 (PMC13154238; doi:10.2174/0113892029353221251120094444)
Supplement: Supplementary file 1 [file CG-26-6-530_SD1.pdf]

Supplementary Material

Improving the Aroma of Millets by Targeting the *Betaine Aldehyde Dehydrogenase 2* Gene: A Promising Approach for Popularising Millet Foods Worldwide

T.P. Ajeesh Krishna[1,#], Mathew Veena[2,#], Theivanayagam Maharajan[1], Jos T. Puthur[2,*] and Stanislaus Antony Ceasar[1,*]

[1]*Division of Plant Molecular Biology and Biotechnology, Department of Biosciences, Rajagiri College of Social Sciences (Autonomous), Kochi, Kerala, 683 104, India;* [2]*Plant Physiology and Biochemistry Division, Department of Botany, University of Calicut, Calicut University Campus, Malappuram, Kerala, 673 635, India*

Supplementary file 1. The protein sequence of BADHs of foxtail millet, finger millet, sorghum and rice used for clustal alignment. The BADHs protein sequences were collected from the Phytozome (https://phytozome-next.jgi.doe.gov/) database. The highlighted (red, pink and green) sequences are believed to be possible essential for the functional activity of the *BADH2* genes in millets and rice.

1. Foxtail millet

>SiBADH1 (Seita.7G127300)
MAAPATVPLRQLFVDGEWRAPAQGRRLPVINPATEAQIGEIPAGTAEDVEAAVAAARAALRRNRGRDWARAPGAVRAKYLRAI
AAKIIERKPELAKLEALDCGKPYDEAAWDMDDVAGCFEYFADQAEALDKRQNSPVTLPMETFKCHLRREPIGVVGLITPWNY
PLLMATWKVAPALAAGCTAVLKPSELSSVTCLELGDVCKEVGLPSGVLNIVTGLGPDAGAPLAAHPDVDKVSFTGSFETGKK
IMAAAAPMVKPVTLELGGKSPIVVFDDVDIEKAVEWTLFGCFWTNGQICSATSRLLVHTKIAKEFKERMVAWSKNIKVSDPLE
DGCRLGPVVSEGQYEKIKKFISNAKTEGATILTGGVRPAHLEKGYYLEPTIITDVSTSMEIWREEVFGPVLCVYEFSTEDEA
IELSNDTHYGLAGAVISNDRERCQRLAEEIDAGCIWVNCSQPCFFHAPWGGNKRSGFGRELGEGGIDNYLNIKQVTEYLSDEPW
GWYPAPAKM*

>SiBADH2 (Seita.6G151100)
MAAPATVPLRQLFVDGEWRAPAQGRRLPVINPATEAQIGEIPAGTAEDVEAAVAAARAALRRNRGRDWARAPGAVRAKYLRAI
AAKIIERKPELAKLEALDCGKPYDEAAWDMDDVAGCFEYFADQAEALDKRQNSPVTLPMETFKCHLRREPIGVVGLITPWNY
PLLMATWKVAPALAAGCTAVLKPSELSSVTCLELGDVCKEVGLPSGVLNIVTGLGPDAGAPLAAHPDVDKVSFTGSFETGKK
IMAAAAPMVKPVTLELGGKSPIVVFDDVDIEKAVEWTLFGCFWTNGQICSATSRLLVHTKIAKEFKERMVAWSKNIKVSDPLE
DGCRLGPVVSEGQYEKIKKFISNAKSEGATILTGGVRPAHLEKGYYLEPTIITDVSTSMEIWREEVFGPVLCVYEFSTEDEA
IELSNDTHYGLAGAVISNDRERCQRLAEEIDAGCIWVNCSQPCFFHAPWGGNKRSGFGRELGEGGIDNYLNIKQVTEYLSDE
PWGWYPAPAKM*

2. Finger millet

>EcBADH1a (ELECO.r07.4AG0314620)
MAAPPREIPRRGLFIGGGWREPSLGRRLPVINPATEATIGDIPAATAEDVELAVAAARDAFSRNGGAQWSRALGAVRAKFLRA
IATKIKDKKSDLALLETLDSGKPLDESDADMDDVAACFEYYADLAEALDGKQHSPISLPMENFKSYVLKEPIGVVGLITPWNY
PLLMATWKVAPALAAGCTAVLKPSELASLTCLELGAICVEIGLPPGVLNIITGLGPEAGAPLASHPHVDKIAFTGSTETGKRIM
TTAAQMVKPVSLELGGKSPLIVFDDVGDIDKAVEWAMFGIFANGGQVCSATSRLLLHEKIAKQFLDRLVAWTKNIKVSDPLE
EGCRLGSVVSEGQYEKVKKFISTARSEGATILYGGARPQHLRRGFFIEPTIITDVSTSMQIWREEVFGPVVCVKEFSTESEAVE
LANDTPYGLAGAVICTDPERCERITKAIHSGIVWVNCSQPCFVQAPWGGNKRSGFGRELGEWGLDNYMTVKQVTKYCSDEPWG
WYKPPSKL*

>EcBADH1b (ELECO.r07.4BG0345670)
MAAPPREIPRRGLFIGGGWREPSLGRCLPVINPATEATIGDIPAATAEDVELAVAAARDAFSRNGGAQWSRAPGAVRAKFLRA
IATKIKDKKTDLALLETLDSGKPLDESDADMDDVAACFEYYADLAEALDGKQHSPISLPMENFKSYVLKEPIGVVGLITPWNY
PLLMATWKVAPALAAGCTAVLKPSELASLTCLELGAICVEIGLPPGVLNIITGLGPEAGAPLASHPHVDKIAFTGSTETGKRIM
TTAAQMVKPVSLELGGKSPLIIFDDVGDIDKAVEWVMFGIFANGGQVCSATSRLLLHEKIAKQFLDRLVAWTKNIKVSDPLE
EGCRLGSVVSEGQYEKINKFISTARSEGATILYGGARPQHLRRGFFVEPTIITDVSTSMQIWREEVFGPVVCVKEFSTESEAVE
LANDTPYGLAGAVISTDPERCERITKAIHSGIVWVNCSQPCFVQAPWGGNKRSGFGRELGEWGLDNYMTVKQVTKYCSDEPW
GWYKPPSKL*

>EcBADH2a (ELECO.r07.8AG0631100)
MASPAAAVPQRQLFIDGEWRAPALGRRIPVVNPSTEGHIGEIPAGTAEDIDAAVKAARAALKRNRGRDWARASGAVRAKYLRAI
AAKITERKPELAKLEALDCGKPYDEATWDMDDVAGCFEFFAGLAEDLDKKQNSPVSLPLENFKCHLRRDPIGVVGLITPWNYP
LLMATWKVAPALAAGCTAVLKPSELASVTCLELADVCKEVGLPSGVLNIVTGLGPDAGAPLAAHPDVDKVSFTGSSATGQKIM
TAAATMVKPVSLELGGKSPIVVFDDVDIDKAVEWTLFGCFWTNGQICSATSRLLIHTKIAKEFIERMVAWSKNIKVSDPLE
EGCRLGPVVSEGQYEKIKKFVANAKSEGATILTGGVRPKHLEKGYYIEPTIITDVSTSMEIWREEVFGPVLCVKEFSTEDEA
IELAND THYGLAGAVLSADRERCQRVAEEIDAGAIWVNCSQPCFCQAPWGGNKRSGFGRELGDGGLDIYLSVKQITEYTSDEP
WGWYQPPSKM*

>EcBADH2b (ELECO.r07.8BG0660040)
MASPAAAVPQRQLFIDGEWRAPALGRRIPVVNPSTEGHIGEILAGTAEDIDAAVKAARAALKRNRGRDWARAPGAVRAKYLRAI
AAKITERKPELAKLEALDCGKPYDEATWDMDDVAGCFEFFAGLAEDLDKKQNSPVSLPLENFKCHLRRDPIGVVGLITPWNYPL
LMATWKVAPALAAGCTAVLKPSELASVTCLELADVCKEVGLPSGVLNIVTGLGPDAGAPLAAHPDVDKVSFTGSSATGQKIM
TAAATMVKPVSLELGGKSPIVVFDDVDIDKAVEWTLFGCFWTNGQICSATSRLLIHTKIAKEFIERMVAWSKNIKVSDPLE
EGCRLGPVVSEGQYEKIKKFVANAKSEGATILTGGVRPKHLEKGYYIEPTIITDVSTSMEIWREEVFGPVLCVKEFSTEDEAI
ELANDTHYGLAGAVLSADRERCQRVAEEIDAGAIWVNCSQPCFCQAPWGGNKRSGFGRELGDGGLDIYLSVKQITEYTSDEP
WGWYQPPSKM*

3. Sorghum

>SbBADH1 (Sobic.006G109500)
MAPPQTVPRRGLFIGGAWREPCLGRRLPVVNPATEATIGDIPAGTAEDVEIAVAAARDAFSRDGGRHWSRASGAMRANFLRAI
AAKIKDRKSELALLETLDSGKPLDEASADMDDVAACFEYYADLAEALDGKQRSPISLPMENFKSYVLKEPIGVVGLITPWNY
PLLMATWKVAPALAAGCTAVLKPSELASVSCLELGAICMEIGLPPGVFNVITGLGPEAGAPLSSHPHVDKIAFTGSTETGKR
IMTSAAQMVKPVSLELGGKSPLIVFDDIRDIDKAVEWTMFGIFANAGQVCSATSRLLLHEKIAKKFLDRLVAWAKNIKVSDPLE
EGCRLGSVVSEGQYEKIKKFISTARSEGATILYGGARPQHLKRGFFLEPTIITDISTSMQIWREEVFGPVICVKEFRRESEA
VELANDTQYGLAGAVISDDQERCERISKALQSGIIWINCSQPCFVQAPWGGNKRSGFGRELGEWGLDNYMTVKQVTKYCSDE
PWGWYQPPSKL*

>SbBADH2 (Sobic.007G130800)
MATPAMVPLRQLFVDGEWRPPAQGRRLPVVNPTTEAHIGEIPAGTAEDVDAAVAAARAALKRNRGRDWARAPGAVRAKYLRA
IAAKVIERKPELAKLEALDCGKPYDEAVWDMDDVAGCFEYFADQAEALDKRQNSPVSLPMETFKCHLRREPIGVVGLITPWNY
PLLMATWKVAPALAAGCTAVLKPSELASVTCLELADICKEVGLPSGVLNIVTGLGPDAGAPLSGHPDVDKVAFTGSFETGKKI
MAAAAPMVKPVTLELGGKSPIVVFDDVDIDKAVEWTLFGCFWTNGQICSATSRLLIHTKIAKEFNERMVAWAKNIKVSDPLE
EGCRLGPVVSEGQYEKIKKFISNAKSEGATILTGGVRPAHLEKGFFIEPTIITDITTSMEIWREEVFGPVLCVKEFSTEDEA
IELANDTQYGLAGAVISGDRERCQRLSEEIDAGCIWVNCSQPCFCQAPWGGNKRSGFGRELGEGGIDNYLSVKQVTEYISDEP
WGWYQSPSKL*

4. Rice

>OsBADH1 (LOC_Os04g39020)
MAAPSAIPRRGLFIGGGWREPSLGRRLPVVNPATEATIGDIPAATAEDVELAVSAARDAFGRDGGRHWSRAPGAVRAKYLKAI
AAKIKDKKSYLALLETLDSGKPLDEAAGDMEDVAACFEYYADLAEALDGKQRAPISLPMENFESYVLKEPIGVVGLITPWNY
PLLMATWKVAPALAAGCTAVLKPSELASLTCLELGGICAEIGLPPGVLNIITGLGTEAGAPLASHPHVDKIAFTGSTETGKRI
MITASQMVKPVSLELGGKSPLIVFDDVDIDKAVEWAMFGCFANAGQVCSATSRLLLHEKIAKRFLDRLVAWAKSIKISDPLE
EGCRLGSVVSEGQYQKIMKFISTARCEGATILYGGARPQHLKRGFFIEPTIITNVSTSMQIWREEVFGPVICVKEFRTEREAVE
LANDTHYGLAGAVISNDLERCERISKAIQSGIVWINCSQPCFVQAPWGGNKRSGFGRELGQWGLDNYLSVKQVTKYCSDEPY
GWYRPPSKL*

>OsBADH2 (LOC_Os08g32870)
MATAIPQRQLFVAGEWRAPALGRRLPVVNPATESPIGEIPAGTAEDVDAAVAAAREALKRNRGRDWARAPGAVRAKYLRAI
AAKIIERKSELARLETLDCGKPLDEAAWDMDDVAGCFEYFADLAESLDKRQNAPVSLPMENFKCYLRKEPIGVVGLITPWNY
PLLMATWKVAPALAAGCTAVLKPSELASVTCLELADVCKEVGLPSGVLNIVTGLGSEAGAPLSSHPGVDKVAFTGSYETGK
KIMASAAPMVKPVSLELGGKSPIVVFDDVDVEKAVEWTLFGCFWTNGQICSATSRLILHKKIAKEFQERMVAWAKNIKVSDPL
EEGCRLGPVVSEGQYEKIKQFVSTAKSQGATILTGGVRPKHLEKGFYIEPTIITDVDTSMQIWREEVFGPVLCVKEFSTEEEAI
ELANDTHYGLAGAVLSGDRERCQRLTEEIDAGIIWVNCSQPCFCQAPWGGNKRSGFGRELGEGGIDNYLSVKQVTEYASDEP
WGWYKSPSKL*

Supplementary file 2. The gene sequence of BADHs of foxtail millet, finger millet, sorghum and rice were collected from the Phytozome (https://phytozome-next.jgi.doe.gov/) database. The highlighted (red, pink and green) sequences are believed to be possible essential for the functional activity of the *BADH2* genes in millets and rice.

1. Foxtail millet
>*SiBADH1* (Seita.7G127300)
ATGGCCGCGCCGCCGCTGGTCCCGCGCCGCGGCCTCTTCGTCGGCGGGGGATGGAGGGAGCCGTCCCTCGGGCGCCGCCT
CCCCGTCGTCAACCCGGCCACCGAGGCCACCATCGGCGACATCCCGGCGGCCACGGCAGAGGACGTGGAGATCGCGGTCGCGGC
GGCGAGGGATGCGTTCTCGCGCGACGGCGGGAGGCACTGGTCGCGCTCCCCCGGGGCCGTGCGGGCCAAGTTCCTCAGGGCG
ATCGCCGCCAAGATTAAAGATAGGAAATCTGATCTGGCTTTGCTTGAGACACTTGATTCCGGGAAGCCTCTGGATGAAACAG
TTGCAGATATGGATGATGTTGCTGCATGCTTTGAGTACTATGCTGATCTGGCAGAAGCTTTAGACGGGAAACAACATTCACCA
ATCCCTCTACCTATGGAAAATTTCAAGTCCTATATACTCAAAGAACCCATTGGGGTTGTTGGACTGATCACTCCTTGGAACTA
TCCTCTGTTGATGGCTACTTGGAAGGTTGCACCTTCCTTGGCTGCTGGGTGTACAGCTGTGTTAAAGCCATCAGAGTTGGCTT
CTCTGACTTGCTTAGAGCTTGGTGCAATATGCATAGAAGTAGGCCTACCTCCAGGTGTGTTGAACATAATTACTGGTCTGGG
CTCTGAAGCTGGTGCTCCATTATCTTCACATCCCCATGTCGATAAGGTTGCTTTTACTGGAAGTACAGAGACTGGTAAGAAGA
TAATGACTGCTGCTGCCCAAATGGTTAAGCCTGTTTCGTTAGAACTTGGAGGCAAAAGTCCACTTATTGTCTTCGATGACGTT
GACATTGACAAAGCTGTTGAATGGGCCATGTTTGGGATCTTTGCGAATGCTGGTCAAGTCTGCAGTGCTACTTCTCGTCTA
CTTCTGCACGAGAAAATAGCAAAGCAGTTCTTGGATAGATTGGTCGCATGGGCAAAGAATATCAAAATCTCCGACCCACTGGAG
GTAGGCTGCAGGCTGGGGTCTGTTGTCAGTGAAGGGCAGTATGAAAAGATAAAGAAGTTCATCTCAACTGCAAGAAGCGAAG
GTGCCACAATTTTGTATGGCGGTGCCCGACCACAGCACCTCAGAAAAGGGTTCTTTATCGAACCTACAATTATAACAGATGTT
AGTACATCAATGCAAATTTGGCGAGAGGAAGTCTTCGGACCAGTCATCTGCATCAAAGAGTTCAGGACAGAGAGTGAAGCTGT
GGAACTCGCAAATGATACTCACTATGGTTTAGCTGGTGCAGTGATCTCTAATGATGAAGAGAGGTGTGAGCGCATTTCAAAGG
CTCTTCATTCTGGTATTGTTTGGATAAATTGCTCGCAACCAACCTTAGTCCAAGCTCCATGGGGAGGGAACAAGCGGAGCGG
TTTTGGTCGTGAGCTCGGAGAATGGGGCCTTGAGAACTACCTGACCGTGAAGCAAGTCACCAAGTACTGCTCGGATGAGCCA
TGGGGATGGTACCAGCCTCCATCGAAGCTGTAA

>*SiBADH2* (Seita.6G151100)
ATGGCCGCGCCGGCGACGGTCCCGCTGCGGCAGCTCTTCGTCGACGGGGAGTGGCGCGCGCCCGCGCAGGGCCGGCGCCTCCCCG
TCATCAACCCCGCAACCGAGGCCCAGATCGGTGAGATCCCGGCGGGCACGGCGGAGGACGTGGAGGCCGCTGTGGCCGCCGCG
CGGGCGGCGCTCAGGAGGAACCGCGGCCGCGACTGGGCGCGCGCGCCGGGGGCCGTCCGGGCCAAGTACCTCCGCGCCATCG
CCGCCAAGATAATCGAGAGGAAACCTGAGCTGGCTAAGCTAGAGGCGCTTGATTGTGGGAAGCCTTATGACGAAGCAGCATGGG
ACATGGATGATGTTGCTGGCTGCTTTGAGTACTTCGCGGATCAGGCGGAAGCCTTGGACAAAAGGCAAAATTCACCAGTTA
CCCTTCCGATGGAAACTTTTAAATGCCATCTTCGGAGAGAGCCTATCGGAGTTGTTGGGCTTATCACTCCTTGGAACTAT
CCGCTCCTGATGGCTACATGGAAGGTAGCTCCTGCTCTGGCTGCTGGTTGTACAGCTGTGTTGAAGCCATCTGAACTGTCAT
CTGTGACTTGCTTAGAGCTTGGTGATGTCTGTAAAGAAGTCGGTCTTCCTTCTGGTGTCTTGAACATTGTGACTGGTTTAGG
TCCTGATGCTGGTGCTCCTTTGGCAGCGCACCCAGATGTTGACAAGGTCTCTTTTACTGGGAGTTTTGAAACTGGTAAGA
AGATTATGGCAGCTGCAGCTCCTATGGTCAAGCCTGTTACACTGGAACTTGGTGGAAAAAGTCCTATAGTAGTATTTGATGA
TGTTGACATTGAAAAAGCTGTTGAATGGACTCTGTTTGGGTGCTTTTGGACTAATGGTCAGATTTGCAGTGCAACATCTC
GTCTTCTTGTCCATACAAAAATTGCCAAAGAGTTTAAGGAGAGGATGGTTGCATGGTCCAAAAATATTAAAGTTTCAG
ATCCACTTGAAGACGGTTGCAGGCTTGGACCAGTTGTTAGCGAAGGACAGTATGAGAAGATTAAGAAGTTCATATCAA
ATGCTAAAAGCGAAGGTGCTACTATTCTGACTGGGGGTGTTAGACCGGCGCATCTTGAGAAGGGGTACTATCTTGAACCCA
CAATTATTACTGATGTAAGCACATCAATGGAAATTTGGAGGGAGGAAGTCTTTGGTCCAGTCCTGTGCGTTTATGAATTTAG
CACTGAAGATGAAGCCATCGAACTGTCCAACGATACACATTATGGCTTGGCTGGTGCTGTAATTTCCAACGATCGCGAACG
ATGCCAGAGATTAGCTGAGGAGATCGACGCTGGATGTATCTGGGTAAACTGCTCGCAACCCTGCTTTTTCCATGCCCCATG
GGGCGGGAACAAGCGCAGTGGCTTCGGACGCGAGCTCGGAGAAGGGGGCATCGATAACTACCTGAACATCAAGCAGGTCAC
GGAGTACCTCTCTGATGAGCCGTGGGGATGGTACCCGGCCCCCGCCAAGATGTAA

2. Finger millet
>*EcBADH1a* (ELECO.r07.4AG0314620)
ATGGCCGCGCCGCCGCGGGAGATCCCCCGCCGGGGCCTGTTCATCGGCGGCGGGTGGAGGGAGCCGTCCCTCGGGCGCCG
CCTCCCTGTAATCAACCCGGCCACCGAGGCCACCATCGGCGACATCCCGGCGGCCACGGCGGAGGACGTAGAGCTCGCGG
TTGCGGCGGCGCGGGACGCGTTCTCGCGCAACGGCGGGGCGCAGTGGTCGCGCGCCCTTGGGGCCGTGCGGGCCAAGTTCC
TCAGGGCGATCGCCACCAAGATTAAAGATAAAAAATCTGATCTGGCTTTGTTGGAGACACTGGATTCTGGGAAGCCTCTG
GATGAATCAGATGCGGACATGGATGATGTCGCAGCTTGCTTCGAGTATTATGCTGATCTGGCAGAAGCTTTAGATGGGAAA
CAGCATTCACCAATCTCGCTACCTATGGAAAATTTCAAGTCCTATGTGCTCAAAGAACCCATTGGGGTTGTCGGACTGATC
ACTCCTTGGAACTATCCCCTTTTGATGGCAACTTGGAAGGTTGCACCTGCCTTGGCTGCTGGGTGTACAGCGGTGTTAAA
GCCGTCAGAGTTGGCTTCTCTCACTTGCTTGGAGCTTGGTGCAATATGTGTTGAGATAGGCCTACCTCCAGGTGTTTTGAAC
ATAATTACTGGTCTGGGCCCTGAAGCTGGTGCTCCATTAGCTTCACATCCCCATGTGGATAAGATTGCTTTTACTGGAAGT

ACAGAGACTGGTAAGAGGATAATGACGACTGCTGCCCAAATGGTTAAGCCTGTTTCACTAGAGCTTGGAGGTAAAGTCCTCTTA
TTGTCTTTGATGACGTTGGCGACATTGATAAAGCTGTTGAATGGGCCATGTTCGGAATTTTTGCAAATGGTGGTCAAGTCTGC
AGTGCAACTTCTCGTTTACTTCTGCACGAGAAAATTGCAAAGCAATTCTTGGATAGATTGGTTGCATGGACAAAGAATATCAAA
GTCTCAGATCCGCTGGAGGAAGGTTGCAGGCTGGGGTCGGTTGTCAGTGAAGGGCAGTACGAAAAGGTAAAGAAATTCATAT
CAACTGCAAGAAGTGAAGGTGCCACCATTTTGTATGGAGGTGCGCGACCTCAGCACCTCAGAAGAGGGTTCTTTATTGAAC
CTACAATAATAACAGATGTTAGTACATCGATGCAAATTTGGCGAGAGGAAGTCTTTGGTCCGGTCGTCTGTGTTAAAGAATT
CAGTACAGAGAGTGAAGCTGTGGAACTTGCAAATGATACTCCCTATGGTCTAGCTGGTGCCGTGATTTGTACTGATCCGG
AGAGGTGTGAGCGCATTACAAAGGCTATTCATTCAGGTATTGTTTGGGTAAATTGCTCTCAACCATGCTTCGTTCAAGCT
CCATGGGGAGGGAACAAGCGGAGTGGCTTTGGTCGAGAGCTTGGAGAATGGGGCCTTGATAACTACATGACCGTGAAGCAAG
TCACGAAGTATTGCTCAGATGAACCATGGGGATGGTATAAGCCTCCATCGAAGCTGTAA

>*EcBADH1b* (ELECO.r07.4BG0345670)
ATGGCCGCGCCGCCGCGAGAGATCCCCCGCCGGGGCCTGTTCATCGGCGGCGGGTGGAGGGAGCCGTCCCTCGGGCGCTGCCT
CCCTGTAATCAACCCAGCCACCGAGGCCACCATCGGCGACATCCCGGCGGCCACGGCGGAGGACGTGGAGCTCGCGGTCGCGG
CGGCGCGGGACGCGTTCTCACGCAACGGCGGGGCGCAGTGGTCGCGCGCCCCTGGGGCCGTGCGGGCCAAGTTCCTCAGGGCG
ATCGCCACCAAGATTAAAGATAAAAAAACTGATCTGGCTTTGTTGGAGACACTGGATTCTGGGAAGCCTCTGGATGAATCAGA
TGCGGACATGGACGATGTCGCAGCTTGCTTCGAGTATTATGCTGATCTGGCAGAAGCTTTGGATGGGAAACAGCATTCACCAA
TCTCACTACCTATGGAAAATTTCAAGTCCTATGTGCTCAAAGAACCCATTGGGGTTGTCGGACTGATCACTCCTTGGAACTATCC
CCTTTTGATGGCAACTTGGAAGGTTGCACCTGCCTTGGCTGCTGGGTGTACAGCTGTGTTAAAGCCATCAGAGTTGGCTTCTC
TGACTTGCTTGGAGCTTGGTGCAATATGTGTTGAGATAGGCCTACCTCCAGGTGTTTTGAACATAATTACTGGTCTGGGCCCTG
AAGCTGGTGCTCCATTAGCTTCACATCCCCATGTGGATAAGATTGCTTTTACTGGAAGTACAGAGACTGGTAAGAGGATAATGA
CGACTGCTGCCCAAATGGTTAAGCCTGTTTCATTAGAGCTTGGAGGCAAAAGTCCTCTTATTATCTTTGATGATGTCGGCGACAT
CGATAAAGCTGTTGAATGGGTCATGTTTGGAATTTTTGCAAATGGTGGTCAAGTCTGCAGTGCAACTTCTCGTTTACTTCTGCA
CGAGAAAATTGCAAAGCAATTCTTGGATAGATTGGTTGCATGGACAAAGAATATCAAAGTCTCGGATCCGCTGGAGGAAGGTTG
CAGGCTGGGGTCGGTTGTCAGTGAAGGGCAGTACGAAAAGATAAATAAATTCATCTCAACTGCAAGAAGTGAAGGTGCCACCA
TTCTGTACGGAGGTGCGCGACCTCAGCACCTCAGAAGAGGGTTCTTTGTTGAACCTACAATAATAACAGATGTTAGTACATCG
ATGCAAATTTGGCGAGAGGAAGTCTTTGGTCCGGTCGTCTGTGTTAAAGAATTCAGTACAGAGAGTGAAGCTGTGGAACTTGC
AAATGATACTCCCTATGGTCTAGCCGGTGCCGTGATTTCTACTGATCCGGAGAGGTGTGAGCGCATTACAAAGGCTATTCATT
CAGGTATTGTTTGGGTAAATTGCTCTCAACCATGCTTCGTTCAAGCTCCGTGGGGAGGGAACAAGCGGAGTGGCTTTGGTCGA
GAGCTTGGAGAATGGGGCCTTGATAACTACATGACCGTGAAGCAAGTTACGAAGTATTGCTCAGACGAACCATGGGGATGGT
ATAAGCCTCCATCGAAGCTGTAA

>*EcBADH2a* (ELECO.r07.8AG0631100)
ATGGCCTCGCCGGCGGCGGCGGTCCCGCAGCGGCAGCTCTTCATCGACGGCGAGTGGCGCGCGCCGGCGCTCGGCCGCCGCAT
CCCCGTCGTCAACCCCTCCACCGAGGGCCACATCGGCGAGATTCCGGCGGGCACGGCGGAGGACATTGATGCCGCGGTGAAG
GCCGCGCGGGCGGCGCTGAAGAGGAACCGCGGCCGCGACTGGGCCCGCGCATCGGGCGCCGTCCGGGCCAAGTACCTCCGCGC
TATCGCCGCCAAGATAACTGAGAGGAAACCTGAGTTGGCAAAGCTAGAAGCACTTGACTGTGGGAAGCCTTATGACGAAG
CAACATGGGACATGGATGATGTTGCTGGGTGTTTCGAGTTCTTTGCAGGTCTGGCAGAAGACTTGGACAAAAAGCAAAATTC
ACCTGTTTCTCTTCCATTGGAAAATTTTAAGTGTCATCTTCGGAGAGATCCTATTGGTGTAGTTGGGCTAATCACACCTTGG
AACTATCCTCTTCTGATGGCTACATGGAAGGTAGCTCCTGCTCTGGCTGCTGGTTGTACAGCTGTGCTAAAGCCATCTGAGTT
GGCTTCAGTGACTTGCTTAGAGCTGGCAGATGTCTGTAAAGAAGTTGGTCTTCCTTCCGGTGTCTTAAACATTGTGACTGGATT
AGGCCCTGATGCTGGTGCTCCTTTGGCAGCACATCCAGATGTTGACAAGGTATCTTTTACTGGGAGTTCAGCAACTGGTCAAAA
GATTATGACTGCTGCAGCTACTATGGTCAAGCCTGTTTCATTGGAACTTGGTGGGAAAAGTCCTATAGTAGTATTTGATGAT
GTTGACATTGACAAAGCTGTTGAATGGACTCTGTTTGGGTGCTTTTGGACCAATGGTCAGATTTGCAGTGCAACTTCTCGT
CTTCTTATCCATACAAAAATTGCTAAAGAGTTTATTGAGAGGATGGTTGCATGGTCAAAAAATATCAAGGTGTCAGATCCCC
TTGAAGAGGGTTGCAGGCTTGGCCCTGTTGTTAGTGAAGGACAGTATGAGAAGATTAAGAAGTTCGTAGCAAATGCTAAAAGC
GAAGGTGCTACTATTTTGACTGGGGGCGTTAGACCAAAGCACCTTGAGAAAGGATACTATATCGAACCCACGATCATTACTGAT
GTCAGCACATCAATGGAAATTTGGAGGGAGGAGGTCTTTGGTCCAGTCCTTTGTGTTAAGGAGTTTAGTACTGAAGATGAAGC
TATTGAACTGGCTAACGATACTCACTATGGTTTGGCCGGTGCTGTGCTTTCTGCTGATCGTGAGCGATGCCAGAGAGTAGCT
GAGGAGATTGATGCAGGAGCCATTTGGGTGAACTGCTCGCAACCCTGCTTCTGCCAAGCTCCATGGGGCGGGAACAAGCGCAGT
GGATTTGGACGTGAACTCGGAGACGGGGGCCTGGATATCTACCTAAGCGTCAAGCAAATCACGGAATACACCTCTGACGAGC
CGTGGGGCTGGTACCAGCCGCCCTCCAAGATGTAA

>*EcBADH2b* (ELECO.r07.8BG0660040)
ATGGCCTCGCCGGCGGCGGCGGTCCCGCAGCGGCAGCTCTTCATCGACGGCGAGTGGCGCGCGCCGGCGCTCGGCCGCCGCAT
CCCCGTCGTCAACCCCTCCACCGAGGGCCACATCGGCGAGATCCTGGCGGGTACGGCGGAGGACATTGACGCCGCGGTGAA
GGCCGCGCGGGCGGCGCTGAAGAGGAACCGCGGCCGCGACTGGGCCCGCGCGCCGGGCGCCGTCCGGGCCAAGTACCTCCGCG
CTATTGCCGCCAAGATAACTGAGAGGAAACCTGAGTTGGCAAAGCTAGAAGCACTTGACTGTGGGAAGCCTTATGACGAAGCAA
CATGGGACATGGATGATGTTGCTGGGTGTTTCGAGTTCTTTGCAGGTCTGGCAGAAGACTTGGACAAAAAGCAAAATTCACCT
GTTTCTCTTCCATTGGAAAATTTTAAGTGTCATCTTCGGAGAGATCCTATTGGTGTAGTTGGGCTAATCACACCTTGGAACTAT

CCTCTTCTGATGGCTACATGGAAGGTAGCTCCTGCTCTGGCTGCTGGTTGTACAGCTGTGCTAAAGCCATCTGAGTTGGCTT
CAGTGACTTGCTTAGAGCTGGCAGATGTCTGTAAAGAAGTTGGTCTTCCTTCAGGCGTCTTAAACATTGTGACTGGATTA
GGCCCTGATGCTGGTGCTCCTTTGGCAGCACATCCAGATGTTGACAAGGTCTCTTTTACTGGGAGTTCAGCAACTGGTCAAAA
GATTATGACTGCTGCAGCTACTATGGTCAAGCCTGTTTCATTGGAACTTGGTGGAAAAAGTCCTATAGTAGTATTTGATGATGTT
GACATTGACAAAGCTGTTGAATGGACTCTGTTTGGGTGCTTTTGGACCAATGGTCAGATTTGCAGTGCAACTTCTCGTCTTCTT
ATCCATACAAAAATTGCTAAAGAGTTTATTGAGAGGATGGTTGCATGGTCAAAAAATATCAAGGTGTCAGATCCCCTTGAA
GAGGGTTGCAGGCTTGGCCCCGTTGTTAGTGAAGGACAGTATGAGAAGATTAAGAAGTTCGTAGCGAATGCTAAAAGCGAAGGT
GCTACTATTCTGACTGGGGGAGTTAGACCAAAGCACCTTGAGAAAGGATACTATATCGAACCCACGATCATTACTGATGTCAG
CACATCAATGGAAATTTGGAGGGAGGAGGTCTTTGGTCCAGTCCTTTGTGTTAAGGAGTTTAGTACTGAAGATGAAGCTATT
GAACTGGCCAACGATACTCACTATGGTTTGGCCGGTGCTGTGCTTTCTGCTGATCGTGAGCGATGCCAGAGAGTAGCTGAGG
AGATTGATGCAGGAGCCATTTGGGTGAACTGCTCGCAACCCTGCTTCTGCCAAGCTCCATGGGGCGGGAACAAGCGCAGTGGA
TTTGGACGTGAACTCGGAGACGGGGGCCTGGATATCTACCTAAGCGTCAAGCAAATCACGGAATACACCTCTGACGAGCCGT
GGGGCTGGTACCAGCCGCCCTCCAAGATGTAA

3. Sorghum

>*SbBADH1* (Sobic.006G109500)
ATGGCGCCGCCGCAGACGGTCCCCCGGCGCGGCCTCTTCATTGGCGGGGCCTGGAGGGAGCCGTGCCTCGGGCGCCGCCT
CCCCGTCGTCAACCCGGCCACGGAGGCCACCATCGGCGACATCCCGGCGGGCACGGCGGAGGACGTGGAGATCGCGGTC
GCCGCGGCGCGGGACGCGTTCTCCCGCGACGGCGGGAGGCACTGGTCGCGTGCCTCTGGGGCCATGCGAGCCAACTTTCT
CAGAGCGATCGCCGCCAAGATTAAAGATAGGAAATCTGAGCTGGCTTTGCTGGAGACACTTGATTCTGGGAAACCTCTGGA
TGAAGCAAGTGCAGACATGGATGATGTCGCTGCATGCTTTGAGTACTATGCTGATCTGGCGGAAGCTTTAGATGGGAAGCAAC
GTTCACCAATCTCTCTGCCTATGGAAAACTTCAAGTCATATGTACTTAAAGAACCCATTGGGGTTGTTGGACTGATCACTCCTT
GGAACTATCCTCTGTTGATGGCAACTTGGAAGGTCGCACCTGCCTTGGCTGCTGGGTGTACAGCTGTATTAAAGCCTTCAGAA
TTGGCTTCTGTGAGTTGCTTAGAGCTTGGTGCAATATGTATGGAAATAGGCCTACCACCAGGTGTCTTCAATGTAATTACTGGT
CTGGGCCCTGAAGCTGGTGCTCCATTATCCTCACATCCCCATGTGGATAAGATTGCTTTTACTGGAAGTACAGAAACTGGTAA
GAGGATAATGACTTCAGCTGCCCAAATGGTTAAGCCTGTTTCATTAGAGCTTGGTGGCAAAAGTCCTCTTATTGTCTTTGAT
GACATTCGTGACATTGACAAAGCTGTTGAATGGACCATGTTTGGGATCTTCGCAAATGCTGGTCAAGTCTGCAGTGCTACTTC
TCGTCTACTGCTGCATGAGAAAATTGCAAAGAAATTCTTGGATAGATTGGTTGCATGGGCAAAGAATATAAAAGTCTCAGATC
CACTGGAGGAAGGCTGCAGACTGGGTTCAGTTGTCAGTGAAGGACAGTACGAAAAGATAAAGAAGTTCATCTCAACTGCAAG
AAGTGAAGGTGCCACAATTCTGTATGGAGGTGCCCGACCACAGCATCTCAAAAGAGGGTTCTTTCTTGAACCTACAATTAT
AACAGATATTAGTACGTCAATGCAAATTTGGCGAGAGGAAGTCTTTGGACCTGTCATCTGCGTTAAAGAATTCAGGAGAGAG
AGTGAAGCTGTGGAACTTGCAAATGATACTCAGTATGGTTTAGCTGGTGCCGTGATCTCCGATGATCAAGAGAGGTGCGAGC
GCATTTCAAAGGCTCTTCAATCTGGCATTATTTGGATAAACTGCTCGCAACCATGCTTCGTCCAAGCTCCATGGGGAGGGAAC
AAGCGGAGCGGTTTTGGTCGGGAGCTCGGAGAATGGGGCCTTGATAACTACATGACCGTGAAGCAAGTCACCAAGTATTGCTC
GGATGAACCGTGGGGATGGTACCAGCCTCCATCCAAGCTGTGA

>*SbBADH2* (Sobic.007G130800)
ATGGCCACGCCAGCGATGGTCCCGCTGCGGCAGCTCTTCGTCGACGGCGAGTGGCGCCCGCCCCGCGCAGGGCCGCCGCCTCCC
CGTCGTCAACCCCACAACCGAGGCCCACATCGGCGAGATCCCGGCGGGCACCGCGGAGGATGTGGACGCCGCGGTGGCTGCG
GCGCGGGCGGCGCTCAAGAGGAACCGCGGCCGTGACTGGGCGCGCGCGCCGGGGGCCGTCCGGGCCAAGTACCTGCGCGCCATC
GCCGCCAAGGTAATTGAGAGGAAACCTGAGCTGGCTAAGCTAGAGGCACTTGATTGTGGGAAGCCTTACGATGAAGCCGTATGGG
ACATGGATGATGTTGCTGGGTGCTTTGAGTACTTTGCGGATCAGGCAGAAGCCTTGGACAAAAGGCAAAATTCCCCAGTTTCTC
TTCCAATGGAAACTTTTAAATGCCACCTCCGGAGAGAGCCTATTGGGGTAGTTGGGCTGATAACTCCTTGGAACTATCC
TCTCCTGATGGCTACATGGAAGGTAGCTCCTGCTCTGGCTGCTGGTTGTACAGCTGTGCTAAAGCCATCTGAATTGG
CTTCTGTGACTTGCTTAGAGCTTGCTGATATCTGTAAAGAAGTCGGTCTTCCTTCCGGTGTCTTGAACATTGTGACAGGATT
AGGTCCTGATGCTGGTGCTCCTTTGTCAGGGCACCCAGATGTTGACAAGGTCGCTTTTACTGGGAGTTTTGAAACTGGAAAG
AAGATTATGGCAGCTGCAGCTCCTATGGTCAAGCCTGTTACACTGGAACTTGGTGGAAAAAGTCCTATAGTAGTATTTGATGA
TGTTGACATTGACAAAGCTGTTGAGTGGACTCTGTTTGGGTGCTTTTGGACCAATGGTCAGATTTGCAGCGCAACATCTCGTCTT
CTTATCCATACAAAAATTGCTAAAGAATTTAATGAGAGGATGGTTGCATGGGCCAAAAATATTAAGGTTTCGGATCCACTTGAA
GAGGGTTGCAGACTTGGGCCAGTTGTTAGTGAAGGACAGTATGAGAAGATTAAGAAGTTCATATCGAATGCCAAAAGCGAA
GGTGCTACTATTCTGACCGGAGGTGTTAGACCGGCGCATCTTGAGAAGGGGTTCTTTATTGAACCAACAATTATTACTGATA
TCACCACATCAATGGAAATCTGGAGGGAGGAAGTCTTTGGTCCAGTCCTGTGTGTTAAAGAATTTAGCACTGAAGATGAAGC
CATTGAACTGGCCAACGATACACAGTATGGTTTGGCTGGTGCTGTAATTTCTGGTGATCGTGAGCGCTGCCAGAGATTATCT
GAGGAGATCGATGCTGGATGTATCTGGGTAAACTGCTCACAACCCTGCTTCTGCCAAGCTCCCTGGGGTGGGAACAAGCGCA
GTGGATTTGGACGTGAGCTTGGAGAAGGGGGCATTGATAACTACCTGAGCGTCAAGCAAGTCACGGAGTACATCTCTGATGAG
CCGTGGGGTTGGTACCAATCCCCCTCCAAGCTGTAA

4. Rice

>*OsBADH1*(LOC_Os04g39020)
ATGGCCGCGCCGTCGGCGATCCCCCGCCGCGGCCTGTTCATCGGCGGCGGGTGGCGGGAGCCGTCCCTCGGCCGCCGCCTCCCC
GTCGTCAACCCGGCCACGGAGGCAACCATCGGTGACATCCCGGCGGCCACGGCGGAGGACGTCGAGCTCGCGGTGTCGGCGGC
GAGGGATGCGTTCGGCCGCGACGGTGGGAGACACTGGTCGCGCGCGCCTGGGGCCGTGCGGGCCAAGTACCTCAAGGCGATCG
CCGCTAAGATTAAAGATAAGAAATCTTATCTAGCTTTGTTGGAGACTCTTGATTCTGGGAAGCCTCTGGATGAAGCAGCTGGGG
ACATGGAGGATGTCGCTGCATGCTTTGAGTATTATGCTGATCTGGCAGAAGCTTTAGATGGGAAACAACGGGCACCAATCTCTC
TACCCATGGAAAATTTTGAGTCCTATGTACTCAAAGAACCCATTGGGGTTGTTGGACTTATCACTCCCTGGAATTATCCTCTG
CTGATGGCTACTTGGAAGGTTGCACCTGCCCTGGCTGCTGGGTGTACAGCTGTATTAAAGCCATCTGAGCTTGCTTCCCTGACA
TGTTTAGAGCTTGGTGGAATATGTGCAGAAATTGGATTACCTCCAGGAGTCTTGAACATAATTACTGGTCTGGGCACTGAA
GCTGGTGCTCCATTAGCTTCACATCCCCATGTGGATAAGATTGCTTTTACTGGAAGCACAGAAACTGGTAAGAGGATAATGAT
TACTGCTTCCCAAATGGTCAAGCCTGTTTCGTTAGAGCTTGGTGGCAAAAGTCCTCTTATTGTCTTTGATGATGTTGATATTG
ATAAAGCTGTTGAGTGGGCCATGTTTGGGTGTTTTGCGAACGCTGGTCAAGTCTGCAGTGCTACTTCTCGTCTACTTTTGCAT
GAGAAAATTGCAAAGCGATTCTTGGATAGGTTGGTTGCATGGGCAAAGAGTATCAAAATCTCAGATCCACTAGAAGAAGGTT
GCAGGCTGGGGTCAGTCGTTAGTGAAGGGCAGTATCAAAAAATAATGAAGTTCATCTCAACAGCAAGATGTGAAGGTGCCACAAT
TCTATATGGGGGTGCCCGACCACAACACCTCAAAAGGGGGTTCTTTATTGAGCCTACTATTATAACAAATGTTAGCACATCAA
TGCAAATTTGGCGAGAGGAAGTCTTTGGACCGGTCATCTGCGTTAAAGAATTTAGGACAGAGCGTGAAGCAGTAGAACTTG
CAAATGATACTCACTATGGTCTAGCTGGCGCTGTGATTTCCAATGATCTAGAGAGGTGCGAGCGCATTTCAAAGGCTATCCAGT
CAGGTATCGTTTGGATAAATTGCTCGCAACCATGCTTTGTTCAAGCTCCATGGGGAGGGAACAAGCGGAGTGGTTTTGGCCGG
GAGCTAGGACAGTGGGGCCTCGATAACTACTTGAGCGTGAAGCAAGTCACCAAGTACTGCTCAGATGAACCATACGGATGGTA
CCGGCCTCCATCCAAGCTGTAG

>*OsBADH2* (LOC_Os08g32870)
ATGGCCACGGCGATCCCGCAGCGGCAGCTCTTCGTCGCCGGCGAGTGGCGCGCCCCCGCGCTCGGCCGCCGCCTCCCCGTCGT
CAACCCCGCCACCGAGTCCCCCATCGGCGAGATCCCGGCGGGCACGGCGGAGGACGTGGACGCGGCGGTGGCGGCGGCGCG
GGAGGCGCTGAAGAGGAACCGGGGCCGCGACTGGGCGCGCGCGCCGGGCGCCGTCCGGGCCAAGTACCTCCGCGCAATCGCGG
CCAAGATAATCGAGAGGAAATCTGAGCTGGCTAGACTAGAGACGCTTGATTGTGGGAAGCCTCTTGATGAAGCAGCATGGGAC
ATGGACGATGTTGCTGGATGCTTTGAGTACTTTGCAGATCTTGCAGAATCCTTGGACAAAAGGCAAAATGCACCTGTCTCTCT
TCCAATGGAAAACTTTAAATGCTATCTTCGGAAAGAGCCTATCGGTGTAGTTGGGTTGATCACACCTTGGAACTATCCTCT
CCTGATGGCAACATGGAAGGTAGCTCCTGCCCTGGCTGCTGGCTGTACAGCTGTACTAAAACCATCTGAATTGGCTTCCGTGA
CTTGTTTGGAGCTTGCTGATGTGTGTAAAGAGGTTGGTCTTCCTTCAGGTGTGCTAAACATAGTGACTGGATTAGGTTCTGA
AGCCGGTGCTCCTTTGTCATCACACCCTGGTGTAGACAAGGTTGCATTTACTGGGAGTTATGAAACTGGTAAAAAGATTATGGC
TTCAGCTGCTCCTATGGTTAAGCCTGTTTCACTGGAACTTGGTGGAAAAAGTCCTATAGTGGTGTTTGATGATGTTGATGTT
GAAAAAGCTGTTGAGTGGACTCTCTTTGGTTGCTTTTGGACCAATGGCCAGATTTGCAGTGCAACATCGCGTCTTATTCTTCAT
AAAAAAATCGCTAAAGAATTTCAAGAAAGGATGGTTGCATGGGCCAAAAATATTAAGGTGTCAGATCCACTTGAAGAGGGTT
GCAGGCTTGGGCCCGTTGTTAGTGAAGGACAGTATGAGAAGATTAAGCAATTTGTATCTACCGCCAAAAGCCAAGGTGCTAC
CATTCTGACTGGTGGGGTTAGACCCAAGCATCTGGAGAAAGGTTTCTATATTGAACCCACAATCATTACTGATGTCGATACA
TCAATGCAAATTTGGAGGGAAGAAGTTTTTGGTCCAGTGCTCTGTGTGAAAGAATTTAGCACTGAAGAAGAAGCCATTGAATT
GGCCAACGATACTCATTATGGTCTGGCTGGTGCTGTGCTTTCCGGTGACCGCGAGCGATGCCAGAGATTAACTGAGGAGATCG
ATGCCGGAATTATCTGGGTGAACTGCTCGCAACCCTGCTTCTGCCAAGCTCCATGGGGCGGGAACAAGCGCAGCGGCTTTGGA
CGCGAGCTCGGAGAAGGGGGCATTGACAACTACCTAAGCGTCAAGCAAGTGACGGAGTACGCCTCCGATGAGCCGTGGGGA
TGGTACAAATCCCCTTCCAAGCTGTAA
